# Genome Re-Sequencing Reveals the Host-Specific Origin of Genetic Variation in *Magnaporthe* Species

**DOI:** 10.3389/fgene.2022.861727

**Published:** 2022-05-16

**Authors:** Jinbin Li, Lin Lu, Qun Wang, Zhufeng Shi, Chengyun Li, Zhixiang Guo

**Affiliations:** ^1^ Yunnan Key Laboratory of Green Prevention and Control of Agricultural Transboundary Pests, Agricultural Environment and Resource Research Institute, Yunnan Academy of Agricultural Sciences, Kunming, China; ^2^ Flower Research Institute, Yunnan Academy of Agricultural Sciences, Kunming, China; ^3^ The Ministry of Education Key Laboratory for Agricultural Biodiversity and Pest Management, Yunnan Agricultural University, Kunming, China

**Keywords:** rice blast, *Magnaporthe oryzae*, genome resequencing, indels, host-specific origin, avr genes

## Abstract

Rice blast is caused by *Magnaporthe oryzae (M. oryzae)*, which is considered one of the most serious pathogens of rice around the globe. It causes severe losses owing to its proven capability to disrupt the host resistance. Recently, its invasion of new hosts like the *Musa* species or banana plants has been noticed. To understand the possible level of genetic variation, we sequenced the genomes of eight different isolates of the *Magnaporthe* species infecting rice, *Digitaria* (a weed), finger millet, *Elusine indica,* and banana plants. Comparative genomic analysis of these eight isolates with the previously well-characterized laboratory strain *M. oryzae* 70-15 was made. The infectivity of the newly isolated strain from *Musa* species suggested that there is no resistance level in the host plants. The sequence analysis revealed that despite genome similarities, both the banana and *Digitaria* isolates have relatively larger genome sizes (∼38.2 and 51.1 Mb, respectively) compared to those of the laboratory reference strain *M. oryzae* 70-15 (∼37 Mb). The gene contraction, expansion, and InDel analysis revealed that during evolution, a higher number of gene insertions and deletions occurred in the blast fungus infecting *Digitaria* and banana. Furthermore, each genome shared thousands of genes, which suggest their common evolution. Overall, our analysis indicates that higher levels of genes insertion or deletions and gain in the total genome size are important factors in disrupting the host immunity and change in host selection.

## Introduction

Rice blast is considered the most disastrous disease of rice owing to its global distribution (in more than 80 countries on all continents) and destructiveness under favorable conditions. It is caused by *M. oryzae* (also known as *Pyricularia oryzae*), which is a filamentous ascomycete fungus. Rice blast is usually responsible for about 10–30% of total rice yield losses annually (Wilson and Talbot, 2009; Ashkani et al., 2015; Sakulkoo et al., 2018). However, under favorable conditions, *M. oryzae* can destroy entire rice plants within 15–20 days post-infection leading up to 100% yield losses subsequently resulting in total crop failure (Musiime et al., 2005; Skamnioti and Gurr, 2009). It is reported that rice blast accounts for the annual rice production losses of above 50 million tonnes globally which are sufficient enough to feed 210–740 million people (Boddy, 2016). Since rice is a staple food, and provides almost 25% of the calories of the daily human diet, its decreased yield because of rice blast is a serious threat to global food security (Asibi et al., 2019).

Currently, synthetic fungicides are commonly used for the control of rice blast, but the use of these chemicals is gradually being reduced to ensure sustainable food production. Fungicides have attendant problems of cost, development of fungal resistance, and damage to ecosystems. Therefore, the use of resistant cultivars, antagonistic phylloplane bacteria, and fungi is also being tested as an alternative option. But the major issue with *M. oryzae* is its rapid ability to overcome resistance based on R-specific genes (Kiyosawa, 1982; Zeigler et al., 1994; Orbach et al., 2000). Understanding the pathogen’s biology is a prerequisite for developing disease control strategies and in the case of *M. oryzae*, it is more crucial to understand its biology as its host range is extended to wheat which is now an urgent problem in South America (Boddy, 2016). The spores of fungus have the inherent adhesive capability for their attachment to the hydrophobic surface of the leaves (Nilson and Talbot 2009). The molecular studies revealed that the Mpg1 gene coded by the fungal genome is responsible for the attachment of the fungus on the plant surface (Talbot et al., 1993). Once the fungus invades the host plant, it can change the hormonal activities of the host cells for its own aggressive growth.

The first genome sequence of *M. oryzae* for strains 70-15 was published in 2005 and now there are over 30 genomes available from different strains around the world. The genome is composed of seven chromosomes and has a size of ∼40 Mb. Its genome regulates the expression of about 1,500 proteins, many with unknown functions, but certainly, there are some effector proteins among them (Xu et al., 2007). Many novel genes affecting various metabolic networks have been identified in different isolates of the *M. oryzae.* Over 20% (2,154) of genes are differentially expressed, mostly up-regulated during the processes prior to invasion (Oh et al., 2008). Temporal analysis of the transcriptome during infection, together with gene replacement experiments, identifies virulence-related genes required for successful invasion. For example, avirulence in some strains of *M. oryzae* has been traced to the loss of function in several specific genes: two of these encode proteins in signaling pathways linked to G-protein-coupled receptors, both of which are required for appressorium formation (Boddy, 2016).

The natural resistance against rice blast fungus has been introduced into the rice cultivars, which is durable and long-lasting (Ashkani et al., 2014: Sharma et al., 2012). Due to the availability of complete genome sequences and studies on the interaction of the rice blast fungus with its hosts, the *Magnaporthe* species appeared as a model organism for genetic studies. The evolution of rice blast fungus is due to point mutations and insertions or deletions of complete genes (Fudal et al., 2009; Van de Wouw et al., 2010). Due to continuous breeding efforts, the fungal populations usually respond by regaining expelled avirulence genes. The gene-for-gene resistance concept is well established for rice blast fungus. It is a proven fact that deletion or point mutation of certain Avr genes like Avr-Pita is associated with resistance breakdown in the field conditions (Orbach et al., 2000; Zhou et al., 2007; Dai et al., 2010).

Due to its agricultural impact and tractability, *M. oryzae* has become a model fungus for studying host-pathogen interactions, at both the cell biology and molecular levels. To determine the genetic variation among the isolates of *M. oryzae*, we sequenced eight field isolates of this fungus collected from different hosts. We envisaged that a mechanistic understanding of the complex interactions among the pathogen, host, and environment will lead to accurate forecasts of pathogen distribution and greatly advance the management of rice blast for global food security under the present scenario of climate change.

## Results

### Characterization of *Magnaporthe* Fungi Isolated From Rice

**FIGURE 1:**
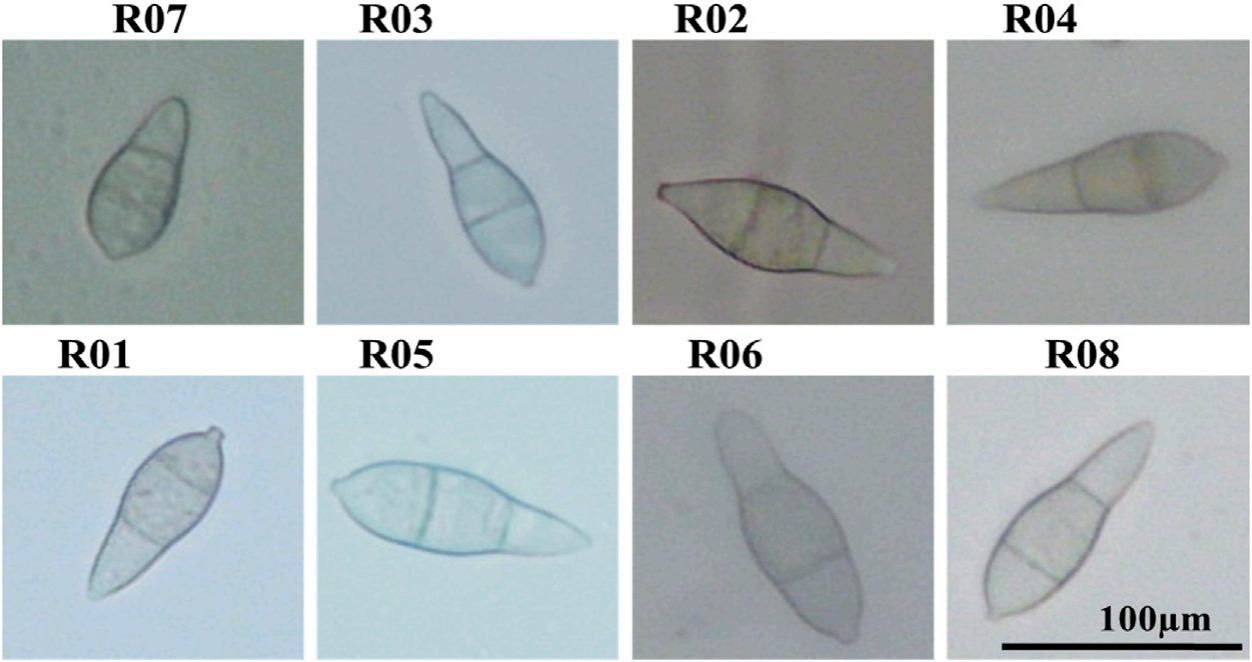
Spore shape of rice blast fungus from a different host. R07, R03, R02, R04, R01, R05, R06, R08 were collected from Banana, *Digitaria sanguinalis*, *Eleusine coracana*, *Eleusine indica*, *Oryza sativa*, *Oryza sativa*, *Oryza sativa*, *Oryza sativa*, respectively*.*

Based on the host’s diversity, morphology, and molecular characterization, eight different strains of rice blast pathogens were identified. The *Magnaporthe* species infecting the above-mentioned hosts including *Oryzae sativa* were germinated for 15 days. The tissue samples obtained from different hosts plants were inoculated on a liquid PDA medium, which resulted in the development of white mycelia. The four strains isolated from *Oryza sativa* were named R01, R05, R06, and R08. While the strains isolated from, *Eleusine coracana, Digitaria sanguinalis, Eleusine indica*, and banana plants were designated as R02, R03, R04, and R07, respectively. The microscopic analysis confirmed the curve-shaped conidium (Figure 1).

**FIGURE 2:**
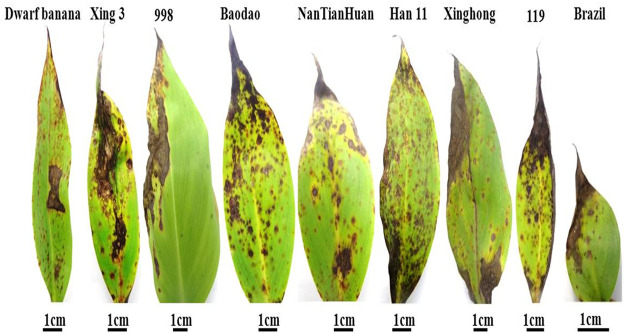
Symptoms of R07 isolate on nine different banana varieties. The symptoms were observed at 12 days post-inoculation.

In order to confirm the pathogenicity of *Pyricularia angulate* (afterward referred to as R07, originally isolated from banana samples), we inoculated it on the leaves of nine different varieties of Banana. Interestingly, all the banana varieties tested here were highly susceptible to the newly isolated *Pyricularia* strain. The infection resulted in angular necrotic lesions on the leaf surfaces (Figure 2). Subsequent confirmation was done through molecular methods. Total extracted DNA was subjected to PCR reaction with ITS1 and ITS4 primers. The ITS region was amplified and further sequencing was performed to confirm the presence of rice blast fungus.

**FIGURE 3:**
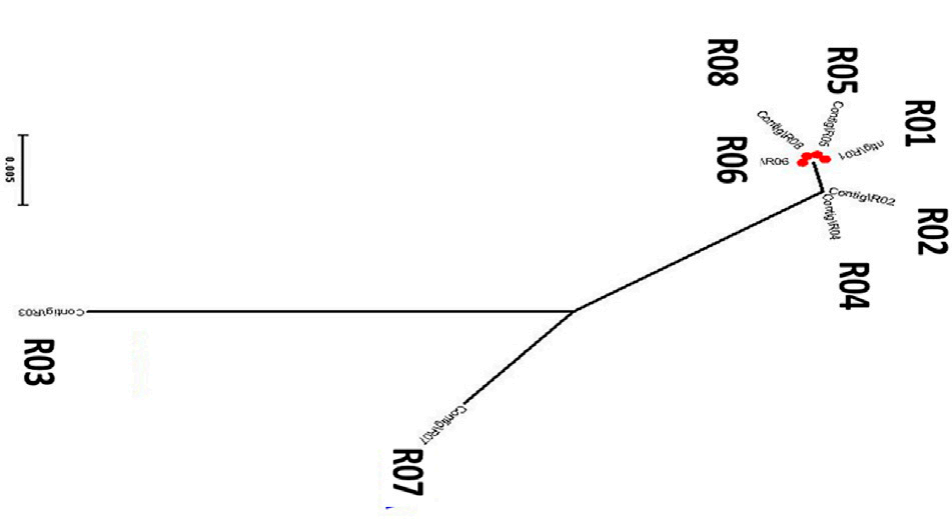
Phylogenetic tree of eight different *Magnaporthe* species isolated from different hosts in Yunnan Province of China. The sequences were derived from the PCR amplified products with ITS1 and ITS4 primers.

The phylogenetic tree constructed from the ITS region suggested that the five isolates (R01, R02, R04, R05, R06, and R08) tightly clustered together. While the two isolates (R03 and R07) isolated from *Digitaria sanguinalis* and banana branched out separately (Figure 3). These observations suggested that R03 and R07 are evolutionary different from other isolates of blast fungus infecting the Gramineae family.

### Disease Reaction on Different Hosts

The four *Magnaporthe* species isolated from rice and four from non-rice plants were inoculated on 14 different hosts of the Gramineae family (Table 1). Interestingly, susceptible disease reaction was noticed for Setaria, Panicum, Coix, Hordeum, and rice fungal isolates against the strains isolated from Rice. Indeed, their infection resulted in typical leaf blast symptoms. While non-rice pathotypes were unable to infect *Oryza* varieties. These results confirmed the hypothesis that *Oryzae sativa* is immune to the blast fungus disease of bananas. Interestingly, none of the Graminae hosts showed susceptibility to the R07 strain isolated from bananas.

**TABLE 1:** Disease reactions of isolates of *Magnaporthe* complex on different gramineous plants.

Variety	Species	Isolates and disease reactions							
		Rice				Non-rice			
		R5	R8	R1	R6	R4	R2	R3	R7
15(Ding)-13	*Setaria italica*	S	S	S	S	S	S	S	R
YuMi2	*Panicum miliaceum*	S	S	S	S	S	S	S	R
YunYi1	*Coix lacryma-jobi* L	S	S	S	S	S	S	S	R
YunKe1	*Hordeum vulgare* var. *nudum*	S	S	S	S	R	S	S	R
YunPi15	*Hordeum vulgare* L	S	S	S	R	R	R	R	R
BaiYan2	*Avena sativa* L	R	R	R	S	S	R	R	R
YunMai52	*Triticum aestivum* L	R	R	S	S	R	R	R	R
Barnyard grass	*Echinochloa crusgalli* (L.) *Beauv*	R	R	S	R	R	R	R	R
YunRui999	*Zea mays* L	R	R	R	R	R	R	R	R
JinNuo10-1	*Sorghum bicolor* (L.) *Moench*	R	R	R	R	R	R	R	R
JinZa22	*Sorghum bicolor* (L.) *Moench*	R	R	R	R	R	R	R	R
JinZa23	*Sorghum bicolor* (L.) *Moench*	R	R	R	R	R	R	R	R
JinZa34	*Sorghum bicolor* (L.) *Moench*	R	R	R	R	R	R	R	R
LTH	*Oryza sativa*	S	S	S	S	R	R	R	R

R = resistant; S = susceptible.

### General Features of Genome Sequencing Assembly

The genomes of blast fungus (R01 to R08) isolated from rice and other hosts were sequenced by the Illumina platform. The Illumina reads were assembled into contigs through SOAPdenovo (V2.04). The assembled genomes consisted of 8,058, 672, 4,611, 993, 2064, 2,242, 550 and 1,081 scaffolds for each strain, respectively (Table 2). The N50 and N90 scaffold lengths for the R07 strain were 251 Kb and 46Kb, respectively (Table 2). Approximately 30% of sequence reads were assembled from more than 1000bp reads for all the strains studied here.

**TABLE 2:** Statistics of various indicators of assembly results.

Sample	R01	R02	R03	R04	R05	R06	R07	R08
No. of all scaffolds	8,058	672	4,611	993	2064	2,242	550	1,081
Bases in all scaffolds (bp)	45,705,591	428,895,122	51,139,513	43,135,702	47,864,486	41,824,542	38,271,459	40,615,511
No. of large scaffords (>1,000 bp)	1,527	273	885	410	716	652	192	464
Bases in large scaffolds (bp)	42,634,984	42,695,629	49,406,385	42,836,693	47,172,095	41,044,594	38,076,148	4,0,311,829
Largest length (bp)	2,802,942	1,897,417	2,596,252	2,532,034	3,182,210	3,257,574	4,847,858	2,805,073
Scaffold N50 (bp)	533,777	720,688	813,585	688,124	759,664	704,994	2,513,868	543,942
Scaffold N90 (bp)	22,515	132,379	41,609	91,109	58,410	52,516	468,507	64,893
G + C content	50.968	49.841	47.806	49.985	50.706	50.943	49.444	51.017
N rate	2.629	2.199	4.868	2.504	1.421	1.735	0.724	2.169
No. of all contigs	11,049	2,957	8,332	3,422	4,192	3,913	1867	2,883
Bases in all contigs (bp)	44,503,682	41,946,281	48,649,628	42,055,534	47,183,856	41,098,713	37,993,995	39,734,454
No. of large contigs (>1,000 bp)	3,070	2,366	3,613	2,544	2,193	2051	1,222	190,888
Bases in large contigs (bp)	40,790,917	41,639,106	46,455,010	41,608,103	46,248,985	40,198,016	37,678,593	39,315,773
Largest length (bp)	226,200	202,057	211,276	196,612	3,069,732	181,031	301,092	190,888
Contig N50 (bp)	34,001	31,883	27,610	31,019	43,189	46,754	71,478	40,967
Contig N90 (bp)	6,298	8,638	5,996	7,880	10,634	9,750	17,314	9,911

To avoid the repetitive genome sequences which were ∼10% in the laboratory reference strain (70-15), the RepeatMasker program was used for genome comparative analysis. As a result, the number of bases in the genomes after masking the repetitive sequences were 45.7, 42.9, 51.1, 43.1, 47.9, 41.8, 38.2, and 40.6 Mb, respectively (Table 2). Whereas the genome size of 70-15 strain is 37.5 Mb (Dean et al., 2005). These results indicate that the size of core genomes of the eight isolates studied here vary significantly. Nevertheless, the genome size of R07 is very close to the reference strain (70-15). Despite genome size differences in different strains, the GC contents across the genomes were centered at 50% (Table 2).

### InDel Length Distribution

InDel length distribution revealed that four isolates of *M. oryzae* from the rice plant (R01, R05, R06, and R08) have the same length distribution of short and long indels while two strains isolated from *Eleusine* showed the same number of indels of different lengths but higher than rice isolates (Supplementary Figure S1). The *M. oryzae* isolated from *Digitaria sanguinalis* showed a quite higher number of short and long inDels as compared to all other fungal isolates. Interestingly, *M. oryzae* isolate from *Digitaria sanguinalis* (R03) exhibited a quite similar InDel distribution with *Pyricularia angulate* isolate from *Musa* spp (R07). In both these species (R03 and R07), the length distribution of InDels was quite higher in non-coding regions as compared to their frequency observed in overall CDS (Supplementary Figure S1). A comparison of InDdel distribution between both isolates (R03 and R07) revealed a relatively higher number of short InDdels (1 to 4 bp) in R03 as compared to that of R07.

### Gene Distribution in the Rice Blast Fungus

Results revealed significant differences in the genome sizes of different strains, and the number of genes also varied in each strain (Table 3). The number of genes was approximately 15.9, 10.6, 13.9, 10.4, 13.7, 11.2, 10.1, and 10.8 K, respectively (Table 3). The average gene size ranged from 1.2 Kb to 1.6 Kb for each strain, which is approximately 0.8 Kb less than the other rice fungus genomes (Fan et al., 2015). On average, genes covered 40% of the genome with the approximate gene density of ∼0.26 genes per Kb which is similar to rice hoppers fungi (Fan et al., 2015).

**TABLE 3:** Gene information statistics.

Sample	R01	R02	R03	R04	R05	R06	R07	R08
Gene num	15,912	10,558	13,972	10,572	13,368	11,244	10,112	10,855
Gene total length (bp)	19,189,195	16,551,272	18,806,884	16,678,618	21,829,110	16,965,085	15,001,029	16,583,180
Gene average length (bp)	1,205.96	1,567.65	1,346.04	1,577.62	1,632.94	1,508.81	1,483.49	1,527.7
Gene density (per kb)	0.35	0.25	0.27	0.25	0.28	0.27	0.26	0.27
GC content in gene region (%)	57.37	57.89	56.34	57.82	56.49	57.62	56.59	57.77
Gene/genome (%)	41.98	38.59	36.78	38.67	45.61	40.56	39.2	40.83
Intergenic region length (bp)	26,516,396	26,338,240	32,332,629	26,457,084	26,035,376	24,859,457	23,270,430	24,032,331
GC content in intergenic region (%)	46.12	44.6	42.43	44.84	45.73	46.26	44.78	46.19
Intergenic length/genome (%)	58.02	61.41	63.22	61.33	54.39	59.44	60.8	59.17

**FIGURE 4:**
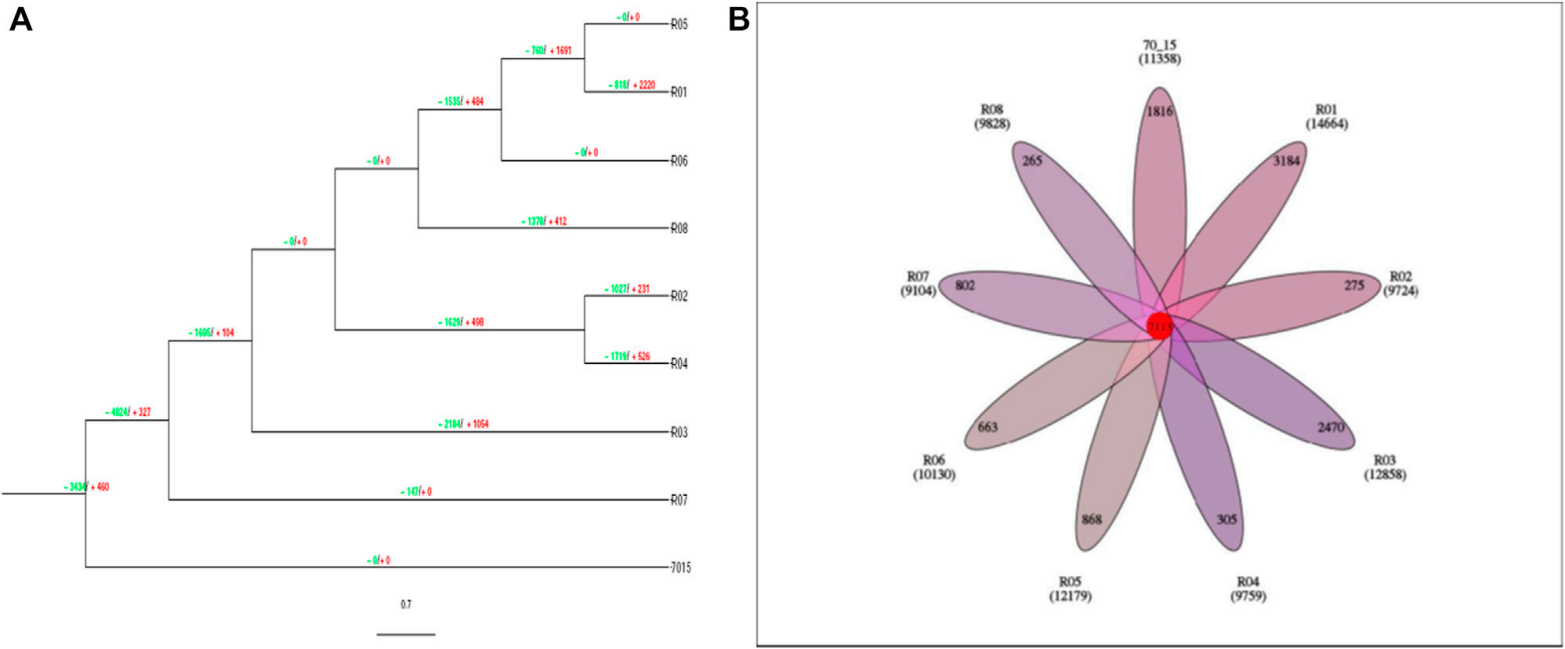
**(A)** Evolutionary branch diagram of gene family expansion and contraction. The tree is rooted on 70-15 strain. The green values represent the reduction while values in red represent the gene expression. **(B)**. The Venn diagram reveals the common ortholog conserved among the rice blast fungus strains. The numbers highlighted in red color represent the common orthologs, while the branch numbers represent the endemic gene families.

As compared with other rice fungal pathogens, these strains have gene families with variable gene expansion and contraction. Just like the reference strain (70-15) which is basal to the phylogenetic tree (Figure 3), R05 and R06 strains had 0 values for gene expansion and contraction, while R07 is only represented by gene contraction (−147). These values indicate that during the course of evolution, the R07 strain deleted gene families (Figure 4A). We also searched for orthologs in these strains. The protein data analysis suggested that these strains share 7,113 proteins with each other representing their common origin (Figure 4B).

**FIGURE 5:**
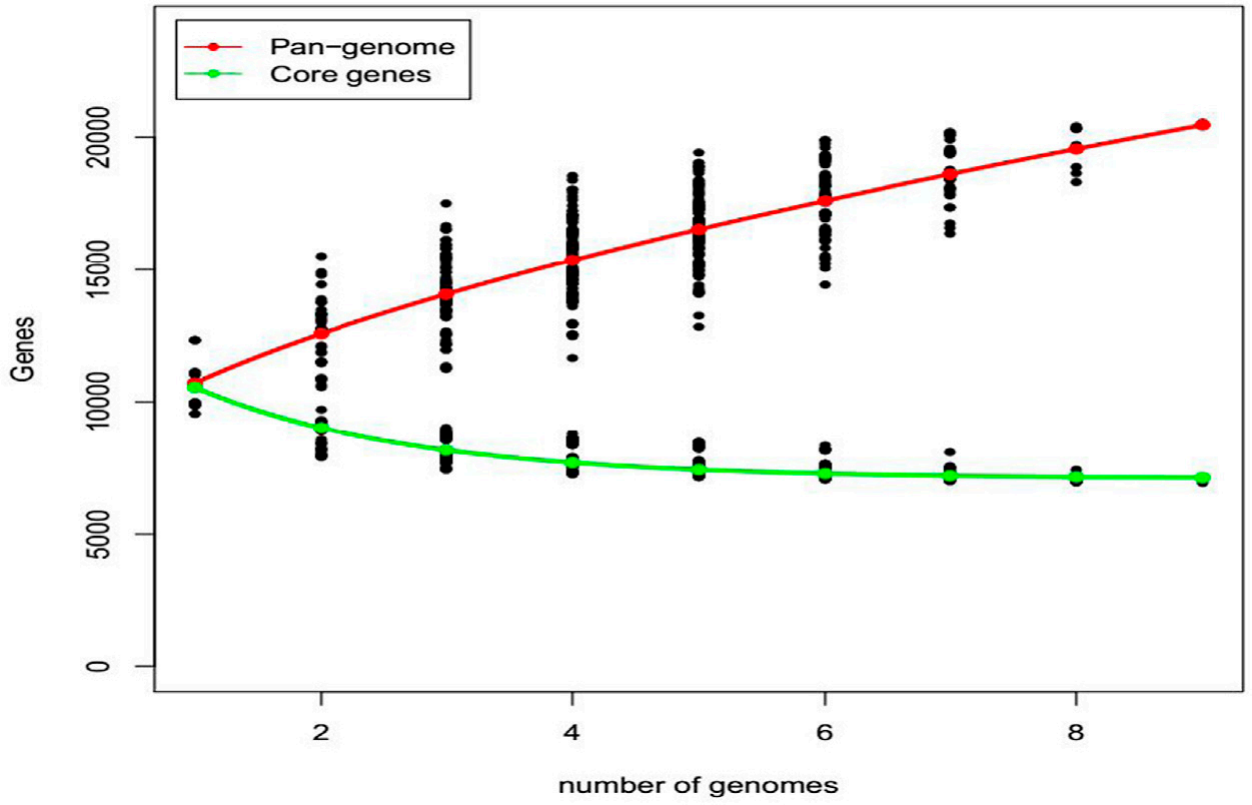
The pan and core genome analysis of eight fungal strains isolated from five different hosts. The red pan-genome curve represents the total number of gene families present in the fungal strains. While the core genome is presented by a green curve showing the conserved gene families.

The contribution of eight fungal strains to the core and pan-genome analysis of the strain 70-15 is presented in Figure 5. All the strains contributed to an increase in the pan-genome. The pan-genome curve demonstrated that all the strains contributed to a sharp increase in gene families. The core genome slightly increased for R01, R02, and R03. While it slightly changed for the R04, R05, R06, R07, and R08 strains. The gene families for the core genome of the R01 strain represented more than 11,000 gene families. However, the strains R03-R08 are presented between ∼6,000 and 8,000 gene families. Overall, there was an increasing trend in the pan-genome plot while a decreasing trend was observed for the core genome plot (Figure 5).

### Presence and Absence of AVR and MAT Genes in Banana Blast Fungus

So far, only 14 Avr genes have been reported from the blast fungus (Huang et al., 2014). In the present study, 14 Avr genes were compared for their presence and absence from the eight different strains sequenced (Supplementary Table S1). Only six Avr genes, namely *AVR1-CO39*, *Avr-Pi54*, *MAT1-1*, *PWL1*, *ACE1*, and *AVR-Pi9* were reported from the banana blast fungus (Supplementary Table S1). Where, the *Avr-Pi54*, *PWL1*, *ACE1*, and *AVR-Pi9* were found conserved among all the strains presented here (Supplementary Table S1). These results are in agreement with the previous reports that Avr genes are conserved and polymorphic in nature (Li et al., 2009). Interestingly, *AVR1-CO39* gene was only present in non-rice host plants. However, *Avr-Pi54*, *PWL1*, *ACE1*, and *AVR-Pi9* were present in all the strains. This data suggests that some gain of Avr genes (*AVR1-CO39*) occurred during the evolution of non-rice fungus. While some other genes were still found conserved among all the fungal strains under investigation. Furthermore, the presence of the MAT1 gene in banana blast fungus (Supplementary Table S2) suggest that sexual reproduction might potential also occur in the field conditions. Interestingly, in all these strains either the *MAT1* or *MAT2* gene was present, which supports the hypothesis that gain or loss of genes is possible due to sexual reproduction.

## Discussion

The comparative fungal genome analysis is important to determine the genetic variation and host selection (Andersen et al., 2011; Xue et al., 2012). The natural genetic variation of rice blast fungus is well established. Rice blast fungus is a worldwide destructive fungal pathogen of many kinds of cereals (Talbot, 2003). Although its first genome sequence was published in 2005, subsequent studies have shown a great array of diversity. Due to the ever-increasing host range, it is important to make evolutionary comparisons based on complete genome sequencing. In this study, genomes of eight different strains of *Magnaporthe* species from five different hosts were sequenced and compared with 70-15 reference genomes. This is the first report on the genome resequencing of *Magnaporthe* species infecting bananas in the Peoples’ republic of China.

Our analysis confirmed the previous reports that *Magnaporthe* species infecting rice are closely related to each other. The sequencing of the ITS region indicated that blast fungus infecting banana is distinct from rice infecting species (Dean et al., 2005). The host pathogenicity test demonstrated that *Magnaporthe* isolates of rice are capable of infecting other cereals including millets and rye species. These results were in agreement with the previous findings that due to its capability to overcome the R genes resistance, the rice blast fungus poses a serious threat to global food security (Asibi et al., 2019).

Our pathogenicity test on nine banana varieties revealed that as a whole banana crop is susceptible to *Magnaporthe* isolates. There is a need to further test the banana germplasm for possible variation in resistance against blast fungus. The present study revealed that blast fungus infecting bananas has certain unique genome characteristics. The genome size of banana infecting blast fungus is relatively smaller than the other isolates of rice in this study and the 70-15 strain (∼38 Mb as compared to ∼41 Mb) (Dean et al., 2005; Xue et al., 2012). Similarly, the number of predicted genes in bananas infecting strain was ∼17% less than the reference strain 70-15 (∼10,112 for R07 compared to rice blast 12,440). In contrast, the R03 strain (isolated from *Digitaria*) had ∼11% more genes as compared to the 70-15 strain. Therefore, it can be concluded that several genes in the rice and non-rice fungus isolates may be different, which could have resulted in differential host selection. In the genomes of R03 and R07 strains, a higher number of InDels was observed, which suggests a possible reason for the host switching from rice to non-rice plants. Because sexual reproduction is not observed for *Magnaporthe* fungi, therefore it is plausible that the difference in genome sizes and a number of genes could be the result of strong selection pressure from infect different hosts. The frequent gain and loss of avirulence genes is an indication of adaptive evolution among fungal strains. The higher conservation of the *ACE1* gene is plausible as it encodes a protein involved in the production of secondary metabolites (Fudal et al., 2005). Previous studies have revealed that Avr genes in *M. oryzae* might pass through several spontaneous deletions and insertions to increase their diversity and as a result, the avirulence may be lost or gained (Dai et al., 2010; Huang et al., 2014). The absence of *AVR-Pita* from banana blast fungus was surprising as, it is an important Avr gene and has previously been reported in non-rice fungal diseases (Chuma et al., 2011).

## Materials and Methods

### Source of Fungus, Morphology, and Hosts

The *Magnaporthe* fungus species namely, *M. oryzae* were isolated from four rice plants (designated as R01, R05, R06, and R08), finger millet (R02), *Digitaria sanguinalis* (R03), *Eleusine indica* (R04), and banana plants (R07) from the Yunnan province of China. The mycelium of fungal species was collected and the cultures were confirmed under the microscope (Nikon eclipse 80i, Nikon Corporation, Japan). The *M. oryzae* was inoculated on 11 different Graminae host species to check the differential response of each species. These 11 host species included, *O. sativa, Setaria italic, Panicum miliaceum, Coix lacryma-jobi L., Avena chinensis, Hordeum vulgare L., Avena sativa L., Triticum aestivum L., Echinochloa crusgalli (L.) Beauv., Zea mays L,.* and *Sorghum bicolor (L.) Moench*.

The *pyricularia angulate* (R07) symptoms severity was assessed on banana leaves. Briefly, the R07 strain was cultured on a PDA medium at 26°C (Peng and Shishiyama, 1988). The nine different varieties namely, Dwarf banana, Xing-3, 998, Baodao, NanTianHuan, Han11, Xinghong, 119, and Brazil were inoculated with R07 strain and the symptoms were evaluated and photographed after 12 days of inoculation. The disease reaction of different strains (R01 to R08) was recorded on 11 different hosts. The disease reaction was recorded as Susceptible (S) for angular necrotic spots appear on leaf surfaces, while resistance (R) was recorded for no discernable symptoms.

### Sequencing of Genomes and Assembly

The fungal species *M. Oryzae* and *Pyricularia angulate* were grown on potato dextrose medium and total genomic DNA was isolated using the Omega Fungal DNA Kit according to the manufacturer’s protocol (Margulies et al., 2005). The fungal strains genome was sequenced according to the already established standard procedures of the paired-end library sequencing approach of Illumina GAII sequencing (Masella et al., 2012). The initial sequencing reads were trimmed and shorter reads (less than 20 nt) were removed. The filtered sequencing reads after Illumina sequencing were assembled using SOAPdenovo (V2.04) genome assembler (Luo et al., 2012). The multi-kmer integrated into SOAPdenovo was used to assemble the contigs. The GapCloser was used to reduce the size of gaps present in the scaffolds generated by SOAPdenovoe2.

### Prediction of Genes and the Genome Annotation

The resulting genomes were annotated using the MAKER2 software (Holt and Yandell, 2011). The gene prediction was carried out using MAKER2 having integrated SNAP tool to predict the mRNA coding regions using prior information from NCBI data. The gene functions were predicted using the STRING database (STRING V11.0; https://string-db.org/). Briefly, the annotated genome of *Magnaporthe* and *Pyricularia* were added to the STRING database to know the proteins and their interaction with other proteins. Since, STRING contains protein information for thousands of genes, therefore adding a genome-wide dataset resulted in robust information on genes coding for different proteins in the newly sequenced genomes.

### Genes Function Prediction Through Cluster of Ortholog Groups (COGs/KOGs)

For comprehensive gene annotation studies, we used three different methods, BLASTp, COGs/KOGs, and STRING database. Briefly, BLASTp was used to confirm the annotated genes’ functions already predicted through the STRING database. The COGs and KOGs were further screened to confirm the ortholog genes’ functions in the newly sequenced genomes.

## Conclusion

Overall, the present study indicated that differences in genome sizes and the number of genes of various *Magnaporthe* species can be attributed to insertions/deletions in the chromosomes and the gain or loss of new gene families. These chromosomal variations might result in the selection of different hosts like bananas or *Digitaria* other than rice. It is the first comprehensive genome study of a fungal pathogen infecting banana which is an economically important crop on the global scale.

## Data Availability

The datasets presented in this study can be found in online repositories. The names of the repository/repositories and accession number(s) can be found in the article/Supplementary Material.
