# Supplementary material for: Genome Re-Sequencing Reveals the Host-Specific Origin of Genetic Variation in Magnaporthe Species
Source: Front Genet. 2022 May 16;13:861727. doi: 10.3389/fgene.2022.861727 (PMC9149001; doi:10.3389/fgene.2022.861727)
Supplement: Supplementary file 1 [file DataSheet1.docx]

**Supplementary Data for**

**Genome re-sequencing reveals host-specific origin of genetic variation in *Magnaporthe* species**


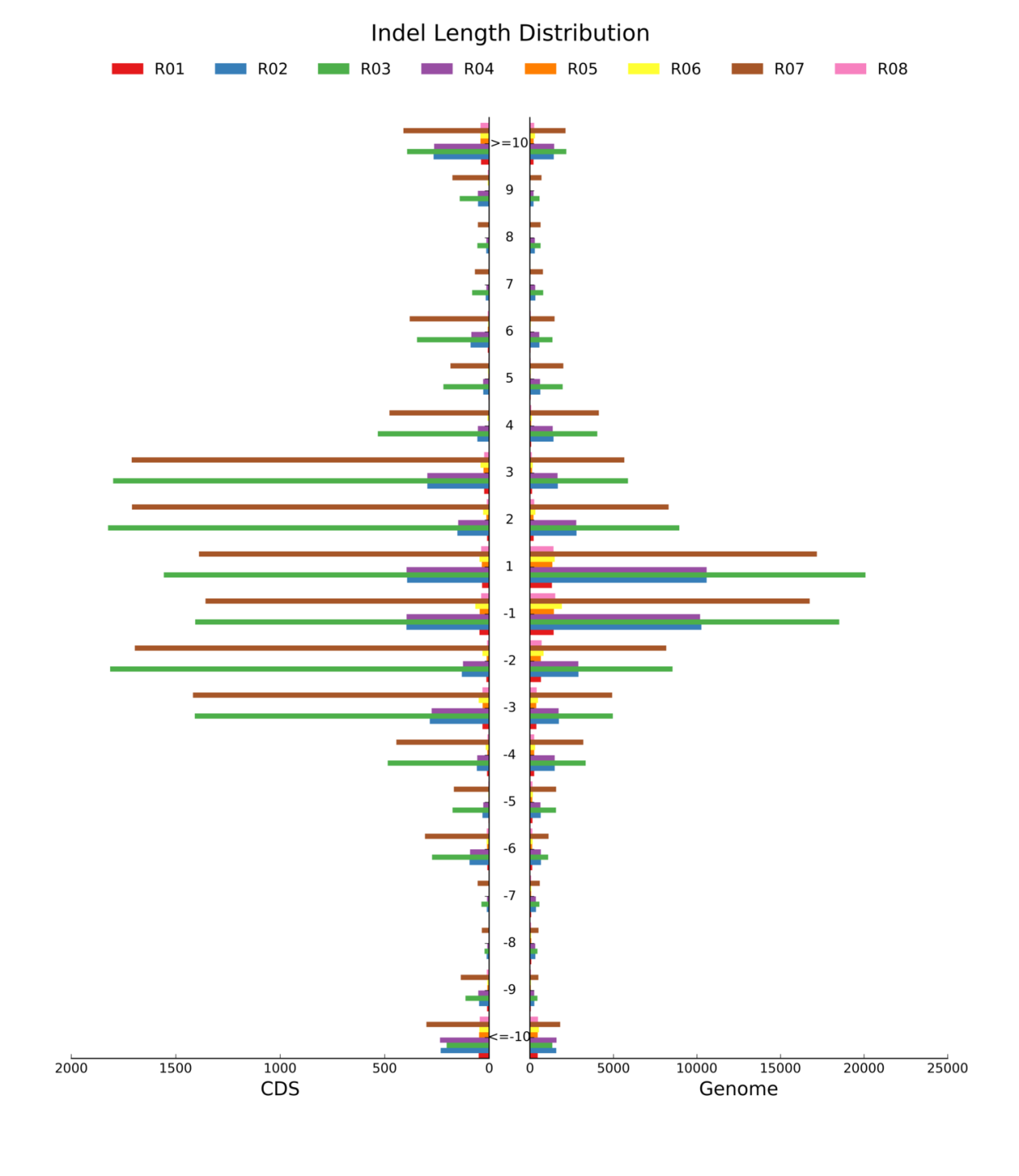


Figure S-I. InDel length distribution map of the whole genome and coding region in different isolates of *Magnaporthe oryzae*

Note: The ordinate is the length of InDel (within 10bp), greater than 0 is Insertion, less than 0 is Deletion, and the abscissa is the corresponding quantity

Table S-1. Distribution of Avirulence gene of *Magnaporthe* species

| *AVR* gene | Genome of isolates | | | | | | | |
| --- | --- | --- | --- | --- | --- | --- | --- | --- |
|  | R01 | **R02** | **R03** | **R04** | R05 | R06 | R07 | R08 |
| *AVR1-CO39* | A | **P** | **P** | **P** | A | A | P | A |
| *Avr-Pi54* | P | **P** | **P** | **P** | P | P | P | P |
| *AVR-Pia* | P | **P** | **P** | **P** | P | P | A | A |
| *AVR-Pii* | A | **A** | **A** | **A** | A | A | A | A |
| *AVR-Pik* | P | **A** | **A** | **A** | A | A | A | A |
| *AVR-Pita* | A | **P** | **P** | **P** | P | P | A | P |
| *AVR-Pizt* | P | **P** | **A** | **P** | P | A | A | P |
|  |  |  |  |  |  |  |  |  |
|  |  |  |  |  |  |  |  |  |
| *PWL1* | P | **P** | **P** | **P** | P | P | P | P |
| *PWL2* | P | **P** | **P** | **P** | P | A | A | P |
| *ACE1* | P | **P** | **P** | **P** | P | P | P | P |
| *AVR-Pi****9*** | P | **P** | **P** | **P** | P | P | P | P |
| *AVR-Pi****b*** | A | **A** | **A** | **A** | A | A | A | A |

Note : P: Present, A:Absent.

Table S-2: Distribution of mating type gene of *Magnaporthe* species

| *MAT* gene | Genome of isolates | | | | | | | |
| --- | --- | --- | --- | --- | --- | --- | --- | --- |
|  | R01 | R02 | R03 | R04 | R05 | R06 | R07 | R08 |
| *MAT1-1* | P | P | A | P | P | A | P | A |
| *MAT1-2* | A | A | P | A | A | P | A | P |

Note : P: Present, A: Absent.
